# Metastatic Thymic Mucoepidermoid Carcinoma: The Diagnostic Challenges and Role of CRTC1/MAML2 Translocation in Accurate Diagnosis and Treatment

**DOI:** 10.1111/1759-7714.70001

**Published:** 2025-01-30

**Authors:** Toshiaki Takahashi, Daniel Graham, Evan Wu

**Affiliations:** ^1^ Department of Medicine, John A. Burns School of Medicine University of Hawaii Honolulu Hawaii USA; ^2^ Hawaii Pacific Health Honolulu Hawaii USA

**Keywords:** comprehensive genomic profiling, mucoepidermoid carcinoma, thymic cancer

## Abstract

Mucoepidermoid carcinoma (MEC) is a subtype of epithelial neoplasms commonly found in salivary glands, but can also be seen in the thymus. Diagnosing MEC of the thymus is sometimes challenging due to its histological similarities with adenosquamous carcinoma (ASC). This case report describes a 64‐year‐old female with a history of metastatic endometrial adenocarcinoma who presented to an oncology clinic with a thymic mass as well as multiple mass lesions in the liver, bone, and abdominal wall. Initially diagnosed as thymic ASC based on histopathology, further genomic profiling revealed a CRTC1/MAML2 translocation, leading to the diagnosis of metastatic MEC of the thymus. Comprehensive genomic testing played a crucial role in distinguishing MEC from ASC. This case highlights the importance of genetic testing in cases of uncertain primary origins and in differentiating between morphologically similar tumors.

## Graphical abstract

We report a case of metastatic mucoepidermoid carcinoma (MEC) of the thymus that was challenging to distinguish from adenosquamous carcinoma due to histological similarities. Genomic testing revealed a CRTC1/MAML2 translocation, confirming metastatic MEC and indicating the critical role of genetic testing in its diagnosis.

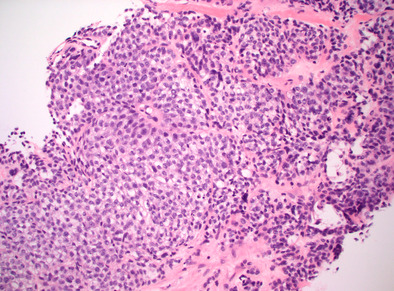

## 1 Introduction

Mucoepidermoid carcinoma (MEC) was first identified in 1945 [1], as a histological subtype of salivary gland tumors, characterized as an epithelial neoplasm originating from the submucosa and is histologically characterized by the presence of squamous cells, mucin‐secreting goblet cells, and intermediate cells. While initially discovered in the salivary glands, this histological pattern has also been identified in the thymus. Currently, thymic MEC is listed in the category of salivary gland‐like carcinoma of the thymus in the World Health Organization (WHO) criteria [2]. Diagnosing thymic MEC can be particularly challenging due to its histological overlap with adenosquamous carcinoma; thus further diagnostic testing has been investigated. CRTC/MAML2 translocation is a specific biomarker for MEC, which has been seen in approximately 60% of MEC cases [3, 4]. Here, we present a case of metastatic thymic MEC, which was diagnosed by a genomic profiling test that identified the CRTC/MAML2 translocation.

### 1.1 Case Description

A 64‐year‐old female with a history of metastatic endometrioid adenocarcinoma was referred to a oncology clinic for further evaluation of her thymic mass. She was diagnosed with Stage IA, Grade 3 endometrioid endometrial adenocarcinoma and underwent robotic‐assisted hysterectomy, bilateral salpingo‐oophorectomy, and lymph node dissection, followed by adjuvant chemotherapy with carboplatin and paclitaxel 1 year prior to the visit.

**FIGURE 1:**
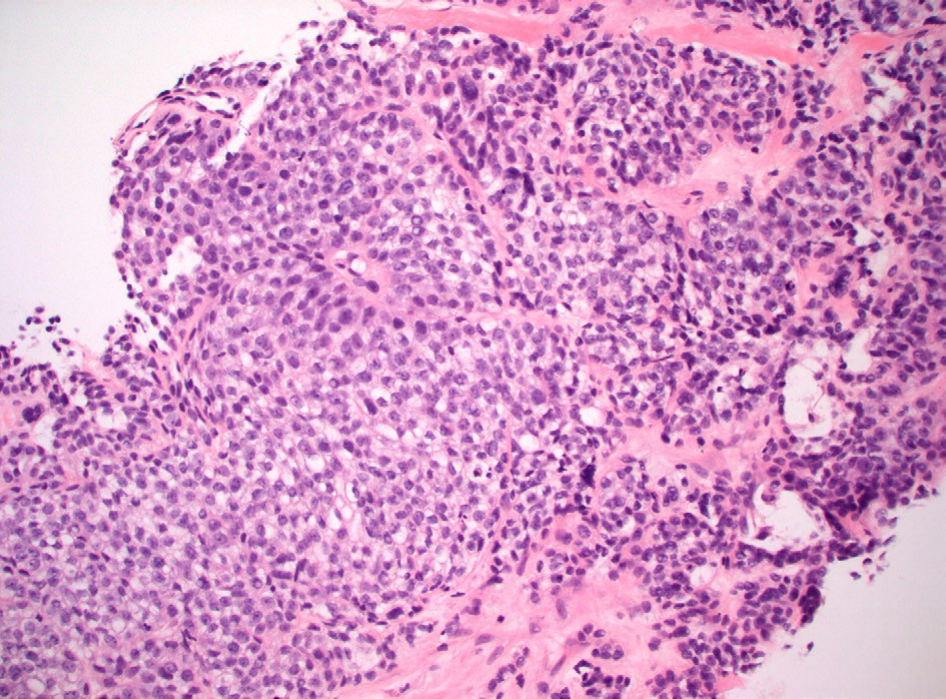
H&E stain of abdominal mass: The tumor is growing in sheets and large nodules, with intervening fibrous bands, and a few scattered cystic/pseudoglandular structures. The overall cytomorphology is somewhat monotonous, with scattered large pleomorphic cells. Tumor cells show minimal to moderate eosinophilic to clear cytoplasm. Overt cytoplasmic mucin is not seen. Chromatin patterns range from slightly open, with small punctate nucleoli, to hyperchromatic and smudgy. Single‐cell necrosis is scattered throughout. Mitotic figures are readily identifiable. These features are non‐specific, and, on their own merit, do not raise the possibility of mucoepidermoid carcinoma. Only post hoc knowledge of the patient's prior diagnosis led to the correct diagnosis of this metastatic deposit.

**FIGURE 2:**
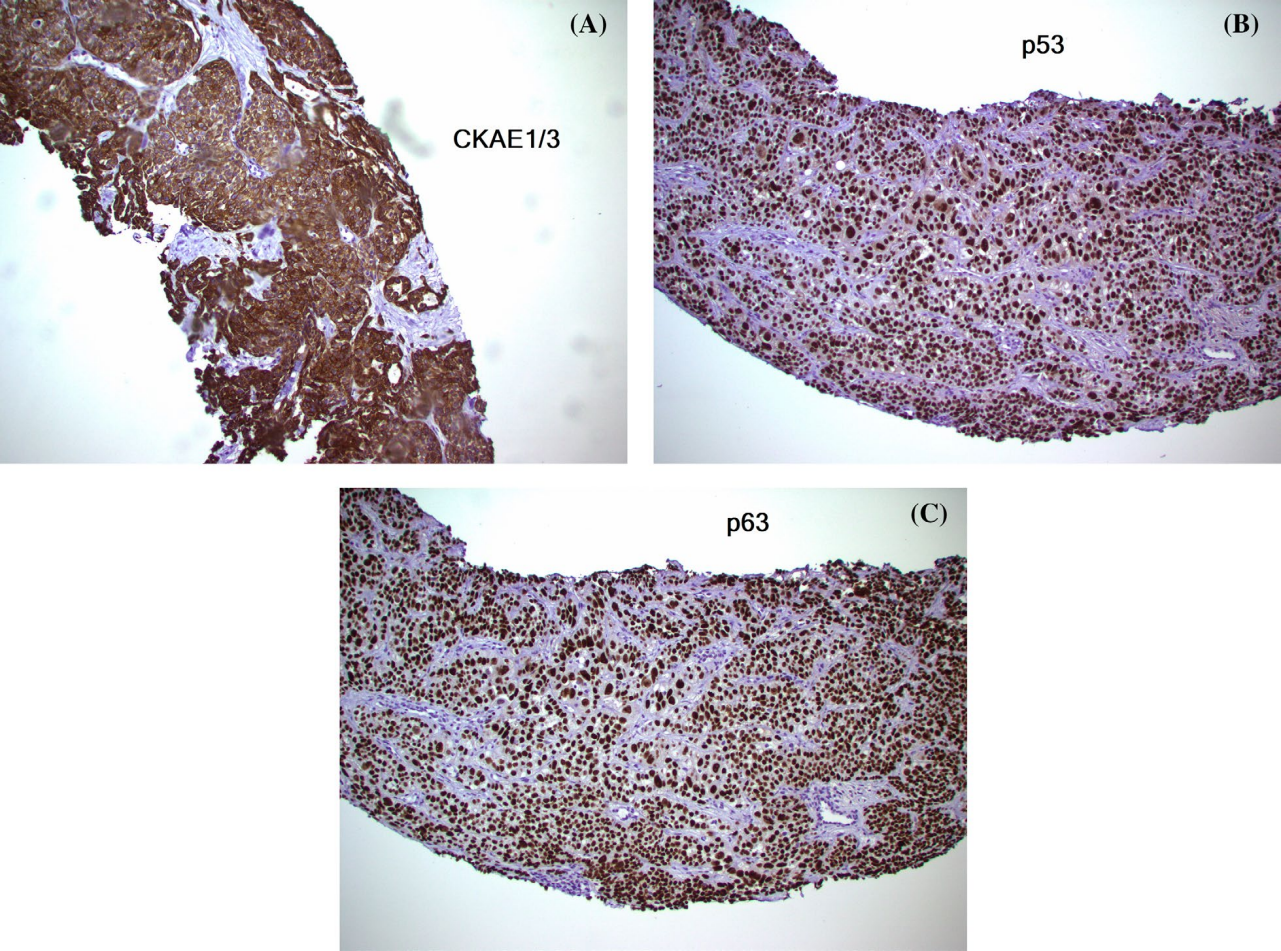
(A) Cytokeratin AE1/AE3 immunostain of abdominal mass: Diffuse strong positive in all tumor cells. (B) p53 Immunostain of abdominal mass: Aberrant overexpression pattern, typically correlating to an underlying TP53 abnormality. (C) p63 Immunostain of abdominal mass: Diffuse strong nuclear positivity in all tumor cells.

She was found to have a 2.8 × 4.6 cm anterior mediastinal mass on CT and underwent a robotic thymectomy in April, 2024. The pathology result was compatible with adenosquamous carcinoma with pericardium and large vessel invasion (Figure 1). Immunohistochemistry (IHC) stains were positive for p63, p40, CD117, and CD5 but negative for PAX8 and ER, which was not matched to the IHC stain of the previous endometrioid adenocarcinoma (Figure 2A–C). Because of different pathological findings, the patient was suspected of having a second primary cancer; however, it was unclear whether this adenosquamous cell carcinoma was the primary lesion or metastatic lesion of a different origin. Further genomic profile testing with Caris Molecular Intelligence Comprehensive Genomic Profiling, a comprehensive cancer testing service offered by Caris Life Sciences (Phoenix, Arizona) that analyzes tumor tissue at the DNA, RNA, and protein level using multiple technologies including next‐generation sequencing (NGS)‐whole exome sequencing (WES) & RNA‐whole transcriptome sequencing (WTS), pyrosequencing, IHC, in situ hybridization (ISH), on the thymus tissue revealed a mutation in BAP1, ex 11, CUL3, CRTC/MAML2, and TP53; which was suggestive of mucoepidermoid carcinoma of the thymus.

Later, she presented to the emergency department with decreased appetite and generalized weakness. She was found to have hypercalcemia at 14.0 mg/dL, complicated by appetite loss and generalized weakness. PTH was 6 pg/mL, and vitamin D was 27 ng/mL. CT revealed a liver mass and bone lesions in the spine, right 11th rib, and pelvis. It also showed soft tissue density within the subcutaneous fat on the upper anterior abdomen. A biopsy of the anterior wall mass showed scattered highly pleomorphic cells with mucin production. IHC was negative for PAX8 and ER, and strongly positive for p63 and p53, with equivocal mucicarmine. These results were consistent with previously diagnosed mucoepidermoid carcinoma and repeat comprehensive genetic testing of the abdominal wall mass again showed a CRTC/MAML2 translocation characteristic of MEC. She was treated with intravenous zoledronic acid and discharged the following day. Based on the pathology results of the abdominal wall biopsy, she was diagnosed with metastatic mucoepidermoid carcinoma with bone, liver, and abdominal wall metastases. She was started on systemic chemotherapy with cisplatin, doxorubicin, and cyclophosphamide but unfortunately had progressive liver failure after 2 cycles and opted for hospice care.

## 2 Discussion

The present case report describes a patient diagnosed with metastatic thymic MEC by detecting CRTC1/MAML2 translocation using comprehensive genomic testing. The initial pathological findings suggested adenosquamous carcinoma (ASC); however, subsequently, the patient was ultimately diagnosed with mucoepidermoid carcinoma of the thymus. Differentiating between MEC and ASC can be challenging due to their overlapping histopathological features, particularly since MEC demonstrates morphological similarities to ASC. Moreover, MEC and ASC share several common sites of occurrence, leading to further diagnostic complexity [5].

In this case, the diagnostic process was complicated by the presence of disseminated metastases in the bone, liver, and abdominal wall, raising questions about the origin of the primary tumor. The possibility of endometrial cancer had been ruled out based on the IHC stain; the possible clinical scenarios included metastasis of an ASC from another organ, metastasis from an endometrial carcinoma, or a primary thymic ASC. The typical metastatic pattern of thymic carcinoma is the local invasion of contiguous mediastinal structures which accounts for 80%, whereas 40% of cases show metastatic spread to bones, lungs, pleura, liver, or lymph nodes [6]. In contrast, ASC is frequently found in the lungs, though it has also been reported in the pancreas, cervix, gallbladder, head and neck, and other anatomical sites [5].

The utilization of genomic profiling tests has significantly increased, especially in cases of uncertain primary origin. In this case, the genomic profiling test revealed a CRTC1/MAML2 translocation, a molecular marker uniquely associated with MEC, located at t(11;19) (q12;p13), resulting in a CRTC1/MAML2 fusion transcript [7]. In distinguishing MEC from ASC, the sensitivity and specificity of the CRTC1/MAML2 translocation have been reported to be 60% and 100%, respectively [4]. The diagnosis of thymic MEC was confirmed given the high specificity of the CRTC1/MAML2 translocation on the genomic profiling test. Furthermore, the biopsy of the abdominal wall mass was consistent with metastasis, supporting the diagnosis of primary thymic MEC. Although the recent retrospective analysis did not show any association between the presence of CRTC1/MAML2 translocation and the prognosis of salivary MEC cases [3], the prognostic implications of CRTC1/MAML2 translocation in thymic MEC remain poorly understood. Given several other gene mutations, such as CDKN2A alteration [8], MECT1‐MAML2 and CRTC3‐MAML2 fusion transcripts [9], found to be associated with prognosis such as CDKN2A, the further utilization of comprehensive genomic profiling test needs to be investigated in order to identify other prognostic factors specifically for thymic MEC, to address diagnostic challenges in patients suspected of having MEC but who test negative for the CRTC1/MAML2 translocation, and ultimately to pursue further personalized approach for the optimal treatment strategies.

## 3 Conclusion

This case highlights the diagnostic complexity of differentiating between thymic MEC and ASC due to their overlapping histopathological features, particularly in the setting of multiple metastases. Comprehensive genomic profiling tests play a crucial role in accurately diagnosing metastatic thymic MEC by detecting specific biomarkers such as CRTC1/MAML2 translocation, particularly in cases of uncertain primary origins.

## Author Contributions

**Toshiaki Takahashi:** Drafted the manuscript and collected the data. **Daniel Graham:** Provided clinical expertise and revised the manuscript. **Evan Wu:** Supervised the project and approved the final version of the manuscript.

## Consent

A written informed consent was obtained from the patient to secure permission to publish the clinical history.

## Conflicts of Interest

The authors declare no conflicts of interest.

## Data Availability

The data that support the findings of this study are available from the corresponding author upon reasonable request.
